# Painless Tooth Drawing

**Published:** 1873-03

**Authors:** 


### PAINLESS TOOTH DRAWING.

ANÆSTHESIA, an inquiry into its origin, by the Hon. Truman Smith, Senator in Congress; also, Nitrous Oxide, as a means for producing insensibility during surgical and other operations, by Charles James Fox, M. R. C. S., L. D. S. We are indebted for these useful publications to Dr. J. Q. Colton, the great pioneer of tooth-drawing without pain by the administration of oxygen or laughing gas. Dr. Colton has drawn teeth for over one hundred and sixty thousand persons, without pain, without the patient knowing it; no case of death ever having occurred in his hands, or in the hands of any other person, as a result of breathing oxygen gas. It is no wonder, then, that the doctor is solicitous that his dear friend, Dr. Horace Wells, of Hartford, Conn., should have awarded to his memory the honor of its discovery, and that his family, who were left without means, should have some substantial testimonial from individuals, and from the nation also.

The tragical fate of Dr. Wells, in New York city, was referred to in the July number. The intelligent reader will be interested to know that Congress was very near giving Dr. Morton's family a hundred thousand dollars for making a discovery, the credit of which belongs justly to Horace Wells, and to no other. The facts are as follows: On the 10th of December, 1844, Dr. J. Q. Colton delivered a chemical lecture at Hartford, Conn.; one part of the performance was to administer laughing gas as an amusement; among those taking it were Dr. Wells and Samuel A. Cooley. Mr. Cooley became very violent, and in his struggles "skinned his shins" extensively, which fact Dr. Wells noticed, and after the excitement was over, asked Mr. Cooley if he felt any hurting. Mr. Cooley professed unconsciousness of any injury, but, on pulling up his pantaloons, he discovered that blood was running freely, and that the skin was scraped from the bone of both legs. In an instant, Dr. Wells turned to a gentleman and exclaimed that he believed if a man took oxygen gas, he could have a tooth extracted without pain, and repeated the expression to his wife on their way home after the lecture. Dr. Wells was a practicing dentist of Hartford, Conn., meriting the confidence and respect of all who knew him. The doctor was so carried away with the idea, and had such a strong conviction of its truth, that he expressed to a friend the determination to have the experiment tried on himself the next morning, and have a tooth extracted from his own jaw. When morning came, he sent for Dr. Colton, seated himself in the dental chair, inhaled the gas, and the tooth was drawn. On recovering his consciousness, he exclaimed to Dr. Colton, "A new era in tooth pulling! It didn't hurt me more than the prick of a pin." And thus was discovered one of the most important truths in the world's history, as connected with bodily suffering, and one cannot but regret that the hero of this discovery could not have lived to enjoy the national reward, as well as a world's gratitude, for a boon so inestimable, instead of having died in jail by his own hand, for a mere indiscretion committed under the hallucination of an over inhalation of nitrous oxide. On the 30th day of September, 1846, near two years later, toward evening, a man residing in Boston, came into the office of a surgeon dentist, Wm. T. G. Morton, suffering great pain, asking if he could have a tooth drawn while he was mesmerized. Dr. Morton said he had something better, and, saturating it with sulphuric ether, gave it to the applicant to inhale. He became unconscious almost immediately. It was dark, and Dr. Hayden held the lamp while Dr. Morton extracted a firmly rooted tooth from the jaw of Eben Frost, who recovered in a minute, not knowing that anything had been done to him. This was Dr. Morton's first anæsthetic experiment on a human being, at the suggestion of Dr. Charles T. Jackson, a learned and very distinguished physician and professor of medicine. On the next day steps were taken to procure a patent for the joint discovery of Drs. Jackson and Morton, and said letters were issued November 12, 1846, and expired in 1860, when Dr. Morton applied for an extension of the patent, but Professor Jackson, from a sense of professional honor, and wishing the whole world to have the benefit of the discovery, refused to join in the application; this made a reissue impracticable by law, and the patent expired.

It is a striking coincidence that Dr. Morton lost his life by violence in New York, by being thrown from a carriage in Central Park. A Mr. Tuckerman paid Dr. Morton fifty thousand dollars for an interest in the business in some way, and liberal men in New York contributed eleven thousand three hundred dollars in addition. But Dr. Wells left a wife and son penniless; the mother supported herself and son by the needle until he grew to be able to earn something as a clerk, and is now able to support her comfortably, but who will not say that the national treasury should not dispense a liberal gratuity to a wife and son so deserving, in memory of a husband and father who has contributed to the world one of the greatest benefactions in its history?

